# Prenatal Particulate Matter Exposure Is Associated with Saliva DNA Methylation at Age 15: Applying Cumulative DNA Methylation Scores as an Exposure Biomarker

**DOI:** 10.3390/toxics9100262

**Published:** 2021-10-13

**Authors:** Kelly M. Bakulski, Jonah D. Fisher, John F. Dou, Arianna Gard, Lisa Schneper, Daniel A. Notterman, Erin B. Ware, Colter Mitchell

**Affiliations:** 1 School of Public Health, University of Michigan, Ann Arbor, MI 48109, USA; johndou@umich.edu; 2 Institute for Social Research, University of Michigan, Ann Arbor, MI 48104, USA; jazzfish@umich.edu (J.D.F.); ebakshis@umich.edu (E.B.W.); cmsm@umich.edu (C.M.); 3 College of Behavioral and Social Sciences, University of Maryland, College Park, MD 20742, USA; arigard@umd.edu; 4 Department of Molecular Biology, Princeton University, Princeton, NJ 08544, USA; lweis@princeton.edu (L.S.); dan1@princeton.edu (D.A.N.)

**Keywords:** DNA methylation, air pollution, particulate matter, saliva, biomarker

## Abstract

Exposure in utero to particulate matter (PM2.5 and PM10) is associated with maladaptive health outcomes. Although exposure to prenatal PM2.5 and PM10 has cord blood DNA methylation signatures at birth, signature persistence into childhood and saliva cross-tissue applicability has not been tested. In the Fragile Families and Child Wellbeing Study, a United States 20-city birth cohort, average residential PM2.5 and PM10 during the three months prior to birth was estimated using air quality monitors with inverse distance weighting. Saliva DNA methylation at ages 9 (*n* = 749) and 15 (*n* = 793) was measured using the Illumina HumanMethylation 450 k BeadArray. Cumulative DNA methylation scores for particulate matter were estimated by weighting participant DNA methylation at each site by independent meta-analysis effect estimates and standardizing the sums. Using a mixed-effects regression analysis, we tested the associations between cumulative DNA methylation scores at ages 9 and 15 and PM exposure during pregnancy, adjusted for child sex, age, race/ethnicity, maternal income-to-needs ratio, nonmartial birth status, and saliva cell-type proportions. Our study sample was 50.5% male, 56.3% non-Hispanic Black, and 19.8% Hispanic, with a median income-to-needs ratio of 1.4. Mean exposure levels for PM2.5 were 27.9 μg/m^3^/day (standard deviation: 7.0; 23.7% of observations exceeded safety standards) and for PM10 were 15.0 μg/m^3^/day (standard deviation: 3.1). An interquartile range increase in PM2.5 exposure (10.73 μg/m^3^/day) was associated with a −0.0287 standard deviation lower cumulative DNA methylation score for PM2.5 (95% CI: −0.0732, 0.0158, *p* = 0.20) across all participants. An interquartile range increase in PM10 exposure (3.20 μg/m^3^/day) was associated with a −0.1472 standard deviation lower cumulative DNA methylation score for PM10 (95% CI: −0.3038, 0.0095, *p* = 0.06) across all participants. The PM10 findings were driven by the age 15 subset where an interquartile range increase in PM10 exposure was associated with a −0.024 standard deviation lower cumulative DNA methylation score for PM10 (95% CI: −0.043, −0.005, *p* = 0.012). Findings were robust to adjustment for PM exposure at ages 1 and 3. In utero PM10-associated DNA methylation differences were identified at age 15 in saliva. Benchmarking the timing and cell-type generalizability is critical for epigenetic exposure biomarker assessment.

## 1. Introduction

Air pollution exposure in utero is associated with adverse pregnancy outcomes [1] and postnatal health problems, such as impaired neurodevelopment [2], increased likelihood of autism spectrum disorder [3], and impaired lung function in children [4]. One component of air pollution is particulate matter (PM), which is classified based on the size of the particle. Smaller particles with a diameter less than 2.5 µM (PM2.5) contain primary combustion particles and secondary particles [5]. Larger particles with diameters greater than 2.5 µM and less than 10 µM (PM10) are generally visible and include black carbon, dust, and mechanically generated particles [5]. PM2.5 and PM10 are heterogeneous exposures, and their chemical makeup depends on the exposure source and the distance from the source. PM2.5 and PM10 differ in the depth of lung penetration [6], though both types of PM are capable of crossing the placenta [7] and thus directly impact the developing fetus. Characterizing the molecular consequences of air pollution exposure during the in utero period is critical to understanding environmentally mediated health disparities that emerge early in life and predict lifelong outcomes.

In utero exposure to PM2.5 and PM10 has well-documented associations with infant DNA methylation in cord blood [8]. Many of these previous studies quantitatively measured DNA methylation using the genome-wide Illumina 450 k array [9]. In a meta-analysis of nine cohort studies, an interquartile range increase in PM2.5 exposure (2 µg/m^3^) was associated with 3% lower DNA methylation near the PLXNA4 gene [10]. Similarly, an interquartile range increase in PM10 exposure (5.6 µg/m^3^) was associated with 1% higher DNA methylation near the GNB2L1 gene [10]. The prenatal period is a window of susceptibility for epigenetic changes such as DNA methylation [11]. Indeed, DNA methylation at birth has been shown to be an effective biomarker of prenatal environmental exposures [12]. However, the identification of these DNA methylation signatures of air pollution in childhood and the blood to saliva cross-tissue applicability has not been tested.

The goal of this study is to investigate air pollution DNA methylation biomarkers in childhood and the cross-tissue applicability between biomarkers developed in cord blood to saliva. Specifically, we tested the associations between in utero air pollution exposure and saliva DNA methylation, measured using the Illumina 450 k array, in the Fragile Families and Child Wellbeing Study. We hypothesized that in utero air pollution exposure would be associated with DNA methylation at ages 9 and 15 in the Fragile Families and Child Wellbeing Study.

## 2. Methods

### 2.1. Study Population

The Fragile Families and Child Wellbeing Study is a United States 20-city birth cohort that recruited children born between 1998 and 2000 [13]. Women were randomly selected from hospitals at the birth of the target child. Unmarried mothers were oversampled by a ratio of 3:1, as the original aims of the study were to examine the downstream effects of families who were disproportionately likely to break up and live in poverty, rather than more advantaged and historically privileged family structures. Participants were excluded from enrollment at baseline if they planned to place the child up for adoption, if the father of the baby was not living at the time of birth, if they did not speak English or Spanish sufficiently to complete the interview, if the mothers or babies were too ill for the mother to complete the interview, or if the baby died before the interview could take place. Assessments continued at ages 1, 3, 5, 9, and 15; an additional follow-up is ongoing. Data collection included medical records extraction, in-home assessments, biosample collection, and surveys of the mother, father, primary caregiver, child, and teachers. This cohort has been extensively used to characterize pathways linking family structure, socioeconomic resources, and child as well as family outcomes (Waldfogel et al. 2010). Participants provided written informed consent for the study. The data used in this manuscript were prepared by the Fragile Families and Childhood Wellbeing Study administrators following approval of the manuscript proposal. These secondary data analyses were approved by the University of Michigan Institutional Review Board (IRB, HUM00129826, approved 31 August 2017).

### 2.2. Covariates and Exposure Measures

Demographic variables were derived from maternal self-report questionnaire data at baseline birth and included maternal race/ethnicity (non-Hispanic Black, non-Hispanic White, Hispanic, Other), household income-to-needs ratio, city of birth (to describe the sampling strategy), and child sex (male, female).

Air pollution exposure was estimated by the Fragile Families and Child Wellbeing Study and provided to the manuscript authors, using methods described previously [14]. At the birth interview, mothers reported their current addresses. Addresses were geocoded and assigned a United States census tract according to the 2000 Decennial Census (for more information see https://fragilefamilies.princeton.edu/restricted). Air pollution data were downloaded from the Inter-university Consortium for Political and Social Research (manifest # 27864) and prepared by the RAND Center for Population Health and Health Disparities [15]. Ambient air quality measures of particulate matter (PM2.5 and PM10; µg/m^3^) were obtained from the US Environmental Protection Agency (EPA) Air Quality System database (US EPA 2018), spanning 1998 to 2000 (the years within which the children in the Fragile Families and Child Wellbeing Study were born). Daily PM concentrations per census tract (based on 2000 Decennial Census definitions) were based on a 24 h mean of PM monitors within 60 km of the census tract, weighted by the inverse distance from the tract centroid to the PM monitors (i.e., the nearest PM monitors were assigned a larger weight in the average PM estimate). Exposure levels were matched to the census tract where mothers reported living at the birth of the child. The date of the child’s birth was used to select the three-month exposure date range prior to birth. Average daily PM2.5 and PM10 exposure concentrations during the three months prior to birth for each participant were calculated by the Fragile Families and Childhood Wellbeing Study and they were our primary exposure variables.

In sensitivity analyses, we considered postnatal exposure at ages one and three. Mothers reported residential addresses at both the age one and age three study visits. The Fragile Families and Childhood Wellbeing Study repeated the above calculations to determine average daily PM2.5 and PM10 concentrations for the three months prior to the age one and age three study visits. At other Fragile Families and Childhood Wellbeing Study visits, residential history and exposure levels were not available.

### 2.3. DNA Methylation Measures and Cumulative DNA Methylation Scores

Child saliva samples at the age 9 and 15 home visits were collected in Oragene kits. Biosamples were not available for prior Fragile Families and Childhood Wellbeing Study visits. Saliva DNA was extracted manually following DNA Genotek’s purification protocol using prepIT L2P. DNA was bisulfite-treated and cleaned using the Zymo Research EZ DNA Methylation Kit. Samples were randomized and plated across slides by demographic characteristics. Saliva DNA methylation at ages 9 and 15 was measured using the Illumina HumanMethylation 450 k BeadArray [9], imaged using the Illumina iScan system. All samples were run in a single batch to minimize technical variability.

DNA methylation image data (IDAT) were processed in R statistical software (version 3.5) using the minfi package [16]. The IDAT pairs (*n* = 1811) were read into R and the minfi preprocessNoob function was used to normalize dye bias and apply background correction before the beta matrix was derived. Further quality control was applied using the ewastools [17] package. Samples that were dropped for QC reasons include: >10% of sites have detection *p*-value > 0.01 (*n* = 43), DNA methylation predicted sex discordant with recorded sex (*n* = 20), and abnormal sex chromosome intensity (*n* = 3). CpG sites were removed if they had detection *p*-value > 0.01 in 5% of samples (*n* = 26,830) or were identified as cross-reactive (*n* = 27,782) [18]. Relative proportions of immune and epithelial cell types were estimated from DNA methylation measures using a childhood saliva reference panel [19].

Our primary cumulative DNA methylation scores were estimated by z-score-standardizing participant DNA methylation at each site, weighting the values by the meta-analysis effect estimates [10] for either PM2.5 (*n* = 14 sites) or PM10 (*n* = 6 sites), and taking the sum across all sites for each participant. Methods for obtaining cumulative DNA methylation scores are evolving; thus, as sensitivity measures, we calculated two secondary cumulative DNA methylation scores. First, we used the direct participant DNA methylation levels (not transformed), weighted by meta-analysis effect estimates, and summed for each participant. Second, we mean-centered the participant DNA methylation levels, weighted by the meta-analysis effect estimates, and summed for each participant. All three DNA methylation scores for each exposure (PM2.5 and PM10) were then z-score-standardized within our study sample for interpretability.

### 2.4. Statistical Analyses

All analyses were conducted in R version 4.1.0. The code to complete the analyses is available (https://github.com/bakulskilab). Distributions of covariates were described using mean and standard deviation for continuous variables with count and frequency for categorical variables. Samples with complete data on exposure, DNA methylation, and demographic information were included in the analysis. The included sample was compared to the excluded sample using t-tests for continuous variables and Fisher’s exact test for categorical variables. We described the sample distributions stratified by study visit (age 9 and age 15). We dichotomized exposures at the median for the study sample and tested for bivariate differences in covariates by exposure. We similarly dichotomized DNA methylation scores at the median for the study sample and tested for bivariate differences in covariates by exposure.

First, we considered analyses that were stratified by the study visit (age 9 or age 15) with the DNA methylation measure. In parallel models stratified for each DNA methylation study visit (age 9 or 15) and for each exposure (PM2.5, PM10), we used multivariable linear regression to test cumulative DNA methylation scores for associations with average exposure levels in the three months prior to birth, adjusted for child sex, child age at DNA methylation measure, maternal income-to-needs ratio, maternal marital status, maternal race/ethnicity, and cell-type proportions. We plotted the residuals of this model versus the observed exposure levels in the three months prior to birth.

Second, we considered information from both study visits with DNA methylation measures. Among the subset of participants with observations at both time points, we made scatter plots of the DNA methylation measures by time point and calculated the Pearson correlation among the two measures. When jointly considering both DNA methylation study visits in adjusted analyses, we used mixed-effects regression models, accounting for within-participant measures with a random intercept in the nlme packge [20]. Mixed-effects models were also adjusted for child sex, child age at DNA methylation measure, maternal income-to-needs ratio, maternal marital status, maternal race/ethnicity, and cell-type proportions. We included participants with observations at either time point in these models (did not require observations at both time points). We reported the fixed-effects estimates for an interquartile range increase in the relevant exposure measure, 95% confidence intervals, and *p*-values for the association.

### 2.5. Sensitivity Analyses

To assess the robustness of our findings, we performed several sensitivity analyses. First, we conducted parallel analyses to those described above on the alternative cumulative DNA methylation score calculation approaches (untransformed, centered). Second, we performed analyses consistent with those above that were additionally mutually adjusted for both exposure types. Third, we performed analyses consistent with those above that were additionally adjusted for postnatal air pollution exposure at age one. Fourth, we performed analyses consistent with those above that were additionally adjusted for postnatal air pollution exposure at age three. Fifth, we tested the specificity of the exposure cumulative DNA methylation score by testing the association of a NO_2_ cumulative DNA methylation score with PM2.5 or PM10 exposure, consistent with the methods described above. Sixth, we tested single DNA methylation sites associated with PM10 in prior meta-analysis results in cord blood [10]. We tested DNA methylation levels at these sites (cg00905156, cg06849931, cg15082635, cg18640183, cg20340716, cg24127244) in saliva at age 15 for association with PM10 exposure at birth, adjusted for child sex, child age at DNA methylation measure, maternal income-to-needs ratio, maternal marital status, maternal race/ethnicity, and cell-type proportions. We compared the effect estimates from our findings and prior results [10].

## 3. Results

### 3.1. Study Sample Descriptive Statistics

**Figure 1:**
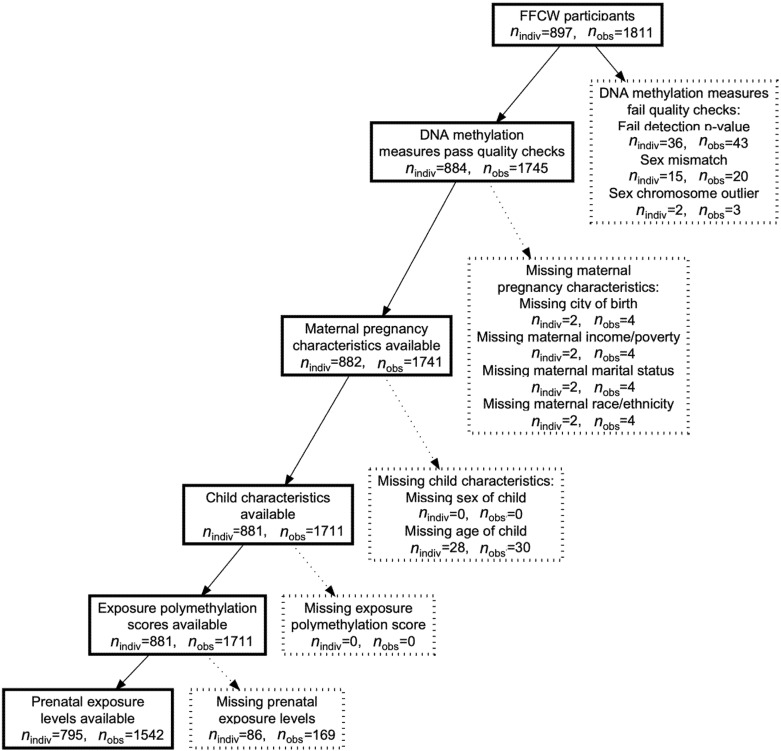
Fragile Families and Child Wellbeing (FFCW) Study sample flowchart. Boxes with solid lines on the left are included observations and individuals. Boxes with dashed lines on the right are excluded observations and individuals. N indiv represents the sample size of individual participants. N obs represents the number of DNA methylation observations. The number of observations can exceed the participants when repeated measures are available.

Among 1811 study samples measured for DNA methylation, information on additional key covariates was available and the DNA methylation data passed quality control for 1542 observations (Figure 1). Included observations were similar to the excluded observations, except the included observations were more likely to be from the age 15 study visit and to be from participants that self-report as non-Hispanic Black (Supplemental Table S1). The included study sample had 749 participants from the age 9 study visit and 793 participants from the age 15 study visit (Table 1). There were 747 participants with measures in both study visits in the included sample. Children in the included sample were 50.5% male, 56.3% non-Hispanic Black, and 19.8% Hispanic, and the mothers had a median income-to-needs ratio of 2.27 at the birth of their child.

PM2.5 concentrations in the three months prior to birth were available for 795 unique participants and PM10 concentrations were available for 736 participants (Supplemental Figure S1). In our analytic sample, average PM2.5 levels in the three months prior to birth ranged from 14.3 to 45.0 μg/m^3^/day with a mean of 27.9 μg/m^3^/day (Supplemental Figure S2A). EPA standards state that 24 h PM2.5 averages should not exceed 35 μg/m^3^ [21]. During the three months prior to birth, 23.7% of the age 15 analytic sample exceeded this standard. In our study, levels of PM2.5 measured prior to birth were correlated with levels of PM2.5 at age 1 (Pearson correlation = 0.54, *p*-value < 2 × 10^−16^, Supplemental Figure S3) and with levels of PM2.5 at age 3 (Pearson correlation = 0.57, *p*-value < 2 × 10^−16^). During the three months prior to birth, average PM10 levels in our sample ranged from 7.5 to 20.2 μg/m^3^/day with a mean of 15.0 μg/m^3^/day (Supplemental Figure S2B). PM10 levels during the three months prior to birth did not exceed EPA standards of a maximum 24 h concentration of 150 μg/m^3^ [21]. In our study, levels of PM10 measured prior to birth were correlated with levels of PM10 at age 1 (Pearson correlation = 0.71, *p*-value < 2 × 10^−16^) and with levels of PM10 at age 3 (Pearson correlation = 0.69, *p*-value < 2 × 10^−16^). Levels of PM2.5 and PM10 measured in the three months prior to birth were correlated (Pearson correlation = 0.2, *p*-value = 7 × 10^−8^).

To calculate cumulative DNA methylation scores for air pollution exposure, we weighted our DNA methylation data using published individual CpG site regression effect estimates from cord blood DNA methylation associated with pregnancy air pollution exposure [10]. We generated separate scores for PM2.5, PM10, and NO2 exposure (using weights from three separate epigenome-wide association tests) and scores were normally distributed within the sample (Supplemental Figure S4). We used three methods to calculate the cumulative DNA methylation scores. Within each pollutant the scores from these three different methods were highly correlated (Pearson correlations ranging from 0.79–1, Supplemental Figure S5). The cumulative DNA methylation scores across pollutants were less highly correlated (Pearson correlation ranging from 0.06–0.71). Among participants with measures at both ages 9 and 15, the cumulative DNA methylation score for PM2.5 was more highly correlated (*r* = 0.55) than the cumulative DNA methylation score for PM10 (*r* = 0.22, Supplemental Figure S6).

### 3.2. Associations between Exposure and DNA Methylation Scores

In bivariate testing, PM2.5 exposure levels and the cumulative DNA methylation score for PM2.5 were not associated in the age 9 subset (*p* = 0.13), nor in the age 15 subset (*p* = 0.48). In mixed-effects regression analyses adjusting for age at DNA methylation sample, child sex, maternal race/ethnicity, maternal income-to-needs ratio, proportion of epithelial cells, and proportion of immune cells, findings were consistent with the bivariate results (Table 2). An interquartile range increase in PM2.5 exposure (10.73 μg/m^3^/day) was associated with a −0.0287 standard deviation lower cumulative DNA methylation score for PM2.5 (95% CI: −0.0732, 0.0158, *p* = 0.20) across all participants. Consistent null findings were observed with cross-sectional multivariable linear regression analyses in the age 9 and age 15 sample subsets and with all three methods for cumulative DNA methylation score calculation.

In bivariate testing, PM10 exposure levels and the cumulative DNA methylation score for PM10 were not associated in the age 9 subset (*p* = 0.22), nor in the age 15 subset (*p* = 0.78). In adjusted mixed-effects regression analyses, we observed associations between PM10 exposure levels and the cumulative DNA methylation score for PM10 (Table 2). An interquartile range increase in PM10 exposure (3.20 μg/m^3^/day) was associated with a −0.1472 standard deviation lower cumulative DNA methylation score for PM10 (95% CI: −0.3038, 0.0095, *p* = 0.06) across all participants. In all participants, consistent negative associations between PM10 exposure levels and the PM10 cumulative DNA methylation score were observed across all three methods for cumulative DNA methylation score calculation. These findings were driven by the age 15 subset, where an interquartile range increase in PM10 exposure was associated with −0.024 standard deviation lower cumulative DNA methylation score for PM10 (95% CI: −0.043, −0.005, *p* = 0.012). We visualized the residuals of the age-stratified models versus the observed PM exposure levels (Supplemental Figure S6).

### 3.3. Sensitivity Analyses

To assess the robustness of our findings, we performed several sensitivity analyses. In all sensitivity analyses, we again observed that prenatal PM2.5 exposure was not associated with the PM2.5 cumulative DNA methylation score. However, we continued to observe that prenatal PM10 exposure was associated with the PM10 cumulative DNA methylation score, particularly in the age 15 sample. First, we repeated the regression analyses with additional adjustment for air pollution levels at age 1 (Supplemental Table S2). The association between prenatal PM10 exposure and age 15 PM10 cumulative DNA methylation score was robust to adjustment for postnatal exposure at age 1 (−0.0302, 95% CI: −0.0556, −0.0047, *p* = 0.020). Second, we repeated the multivariable linear regression analyses with additional adjustment for air pollution levels at age 3 (Supplemental Table S3). The association between prenatal PM10 exposure with the age 15 PM10 cumulative DNA methylation score was robust to adjustment for postnatal exposure at age 3 (−0.0343, 95% CI: −0.0604, −0.0082, *p* = 0.010). Third, we repeated the regression analyses with additional adjustment for prenatal air pollution levels of the other type of particulate matter (Supplemental Table S4). The association between prenatal PM10 exposure with the age 15 PM10 cumulative DNA methylation score was robust to adjustment for prenatal PM2.5 exposure (−0.0231, 95% CI: −0.0424, −0.0038, *p* = 0.019). Fourth, we tested for adjusted associations between prenatal PM2.5 or prenatal PM10 exposure levels with cumulative DNA methylation scores for NO2 (Supplemental Table S5). Prenatal PM10 exposure was associated with the age 15 NO2 cumulative DNA methylation score (0.1271, 95% CI: 0.0520, 0.2022, *p* = 0.0009).

We next attempted to replicate six individual CpG sites previously associated with air pollution exposure in a cord blood meta-analysis at genome-wide significance levels. We observed that DNA methylation at two of these sites in saliva at age 15 was associated with PM10 at birth (Table 3). Specifically, at cg18640183 (associated with the P4HA2 gene) an IQR unit increase in PM10 exposure at birth was associated with 0.119 lower percent DNA methylation (*p* = 0.027). At cg20340716 (associated with the USP43 gene) an IQR unit increase in PM10 exposure at birth was associated with 0.135 higher percent DNA methylation (*p* = 0.015).

## 4. Discussion

In the nationwide, population-based Fragile Families and Child Wellbeing Study birth cohort, we observed that prenatal PM10 exposure was associated with saliva DNA methylation measured at age 15. Previous meta-analyses documented that prenatal air pollution exposure was associated with cord blood DNA methylation at birth [10]. We used effect estimates from these associations to weight measures of saliva DNA methylation at ages 9 and 15 to create cumulative DNA methylation scores for prenatal air pollution exposure. Using these cumulative DNA methylation scores, as well as candidate DNA methylation sites, we observed that average PM10 exposure during the three months prior to birth was associated with DNA methylation differences at age 15 in saliva. Benchmarking the postnatal detection and cell-type generalizability of epigenetic exposure biomarker assessment is critical for its application to epidemiologic applications.

A recent systematic review of prenatal air pollution and infant DNA methylation identified 21 studies focusing on particulate matter [8]. Most of these studies examined candidate genes or global DNA methylation. There have been two prior particulate matter epigenome-wide association studies identified, including one in blood [10] and one in placenta [22]. The particulate matter epigenome-wide association study conducted in blood [10] was done by the PACE consortium and included 1949 participants in the PM10 discovery analysis and 1551 participants in the PM2.5 discovery analysis. This meta-analysis was conducted using cohort data from five European countries (Spain, Netherlands, Belgium, Italy, Greece) and four US cohorts (recruiting from cities nationwide), likely reflecting a wide range of exposure sources. The PACE consortium air pollution findings replicated in an independent cord blood sample and postnatal blood showed persistence of the findings until ages 15 and 16. This study provided the weights for the cumulative DNA methylation scores in our current analysis and identified the candidate DNA methylation sites associated with PM10 exposure for sensitivity analyses in our current study.

In our nationwide study reflecting a wide range of exposures, among age 15 participants, we observed that an interquartile range increase in PM10 exposure (3.20 μg/m^3^/day) was associated with a −0.024 standard deviation lower cumulative DNA methylation score for PM10 (*p* = 0.012). The observed direction of the effect was the opposite of our initial hypothesis (higher exposure would be associated with higher cumulative DNA methylation score). However, the weights used to build our cumulative DNA methylation were from the prior PACE consortium analysis in blood and we measured DNA methylation in saliva, and prior research has shown cross-tissue differences in magnitude and direction of effects for other traits [23]. Similar to the cord blood paper, our saliva samples are estimated to contain a large proportion of immune cells (though likely different proportions or types of immune cells), but our saliva samples also contain DNA from large epithelial keratinocytes from the hard palate [19], and cell-type heterogeneity is a strong predictor of DNA methylation [24]. Among six individual DNA methylation sites that were previously associated with PM10 exposure in cord blood [10], we observed an association in our study at two of those sites (from the P4HA2 and USP43 genes). In addition, in our study we observed an association between PM10 exposure during the three months before birth and DNA methylation measured at age 15, but we did not observe a similar association with DNA methylation measured at age 9. This was also a surprising result as we expected that DNA methylation measures nearer to the time of exposure would have stronger associations. The age 9 study sample had 45 fewer participants than the age 15 study sample, but this alone was unlikely to impact the magnitude of the association. Instead, we hypothesize that there may be greater age-related differences in DNA methylation or DNA methylation measurement error in the sites that contributed to the cumulative DNA methylation score for PM10 exposure. Indeed, among the subset of participants with measurements at both time points, we observed a lower correlation in the cumulative DNA methylation scores for PM10 exposure between ages 9 and 15 (*r* = 0.22) than for the cumulative DNA methylation scores for PM2.5 exposure (*r* = 0.55), and similar lower correlations were observed for the candidate sites associated with PM10 exposure (cg18640183 *r* = 0.35; cg20340716 *r* = 0.27). Of note, the PM2.5 DNA methylation score was based on a larger number of DNA methylation sites (*n* = 14) than the PM10 DNA methylation score (*n* = 6), given the findings from the original meta-analysis, which suggests that scores based on a larger number of DNA methylation sites may be more stable. These findings require replication in additional study populations to determine the reliability of the measures and reproducibility of the associations. We observed associations between prenatal PM10 exposure and saliva DNA methylation at age 15; however, the direction of association was opposite that of prior associations in cord blood at birth and the findings were not consistent with the age 9 sample.

For several additional environmental exposures, epigenetic biomarkers in peripheral tissues (such as blood and saliva) have been shown to be specific and reproducible [25]. The most well-characterized environmental epigenetic biomarker is for cigarette smoke exposure. DNA methylation sites associated with prenatal cigarette smoke exposure have been documented in cord blood meta-analyses [26]. These associations are persistent to age 5 [27] and adolescence [28]. There is also evidence that folate or prenatal vitamin exposure during pregnancy has reproducible DNA methylation associations in cord blood [29,30], though the persistence of these associations postnatally has not yet been tested. In this study, we examined PM, which has a broad exposure and particles can contain multiple types of toxicants that can vary geographically [31]. Our findings may also not be specific to PM10 exposure, as in sensitivity analyses we observed that higher PM10 exposure in the three months prior to birth was associated with a higher cumulative DNA methylation score for NO2. We were surprised that we did not observe an association between PM2.5 and cumulative DNA methylation scores for PM2.5 in this study. Variability in PM components across studies and within our US nationwide study may be part of why we did not observe an association between PM2.5 exposure and cumulative DNA methylation score for PM2.5. The associations between PM2.5 and DNA methylation may be non-persistent or based on acute exposures. Future studies can examine the dose, duration, and composition of PM’s impact on DNA methylation. In other studies, DNA methylation has been associated with children’s health, such as asthma [32], body mass index [33], and attention deficit hyperactivity disorder [34]. Future research in the Fragile Families and Childhood Wellbeing Study may test similar associations between DNA methylation and health outcomes. Prior work demonstrates smoking and folate/prenatal vitamin exposures have reproducible DNA methylation signatures. Additionally, this paper shows air pollution signatures are detectable in saliva, an accessible tissue for epidemiologic research.

Cumulative DNA methylation scores are an emerging area of DNA methylation research. They are an approach within which to apply prior epigenome-wide discovery results to an independent cohort and aggregate epigenome-wide information into a single value. Previous research has documented cumulative DNA methylation score utility as a marker for smoking exposure, which was able to predict prenatal cigarette smoke exposure 30 years later in blood with an area under the curve of 0.72 (95% confidence interval: 0.69, 0.76) in the ALSPAC cohort [35]. Cumulative DNA methylation scores are analogous to polygenic scores, which are widely used in genetic epidemiology [36]. Early findings suggest that cumulative DNA methylation scores for some traits may explain a proportion of the variance comparable to polygenic scores. For example, when predicting body mass index in the Lothian Birth Cohorts, the cumulative DNA methylation score explained 7% of the variance, the polygenic score predicted 8%, and the model containing both predicted 14% [37]. This suggests that the DNA methylation and genetic components for that trait may be independent. Further testing of cumulative DNA methylation scores for additional exposures and traits will be needed to assess the generalizability of these findings.

There are several strengths and limitations of this study. First, the Fragile Families and Child Wellbeing Study is a well-characterized, large, diverse birth cohort with prospective DNA methylation sample collection at two time points. Much of epigenetic epidemiology is cross-sectional and focused on non-Hispanic White participants [38]. Particulate matter exposure levels were quantitated based on residential history, which is standard in the field [39]. Participants may spend considerable time away from home; thus, there is likely measurement error in the exposure estimates, which has been shown to bias estimates towards the null [40]. In addition, US residents are estimated to spend 87% of time indoors [41], and while outdoor PM influences indoor PM exposure levels, buildings can vary in their ability to mitigate outdoor-to-indoor infiltration [42], which would also introduce measurement error in the exposure estimates. Our air pollution exposure estimates were based on outdoor PM levels averaged during the three months prior to birth, which may be confounded with season of birth, an important limitation of our study. Our sensitivity analyses adjusted for air pollution exposure at ages one and three. Unfortunately, exposure measures at the later Fragile Families and Childhood Wellbeing Study visits, including those concurrent with the DNA methylation measures, were not available, thus our DNA methylation analyses were not able to be adjusted for concurrent exposure. The meta-analysis used for our cumulative DNA methylation score weights averaged exposure estimates over the entire pregnancy, which is a larger window of exposure than our study was able to capture. Future studies may separate out DNA methylation signatures specific to trimesters or months of pregnancy to investigate windows of susceptibility. In our study, DNA methylation was measured on a reproducible genome-wide array using methods consistent with prior research. Our cumulative DNA methylation scores were calculated using effect estimates from a large consortium [10] and, importantly, our study sample was independent from the sample that generated the weights [43]. We performed multiple essential sensitivity analyses, including using three methods for cumulative DNA methylation score development, adjustment for postnatal exposure, and adjustment for alternate air pollution exposures. Together, these study design and analytic design elements contribute to rigorous research.

Particulate matter air pollution exposure is associated with global mortality [44] and adverse pregnancy outcomes [1]. In particular, exposure during the in utero period has lasting health effects [2]. Examining the DNA methylation consequences of in utero air pollution is useful to develop biomarkers of air pollution exposure, as well as to document potential molecular intermediates of health effects. Prior research documented in utero air pollution exposure was associated with cord blood DNA methylation. We newly showed that air-pollution-associated DNA methylation differences are detectable at age 15 and that they are detectable in saliva. This study demonstrates postnatal detection and the cross-tissue utility of DNA methylation as a biomarker of air pollution exposure, with important implications for future epidemiology studies.

## Figures and Tables

**Table 1:** Univariate descriptive statistics in the analytic sample of the Fragile Families and Child Wellbeing Study. Participants are grouped by age at DNA methylation assessment. PM: particulate matter.

Characteristic	Overall *n* = 1542	Age 9 Visit *n* = 749	Age 15 Visit *n* = 793	*p*-Value	Number of Observations
**Child Characteristics**					
Sex				0.836	1542
Female	769 (49.9%)	371 (49.5%)	398 (50.2%)		
Male	773 (50.1%)	378 (50.5%)	395 (49.8%)		
Race/ethnicity				0.999	1542
Non-Hispanic White	256 (16.6%)	124 (16.6%)	132 (16.6%)		
Non-Hispanic Black	868 (56.3%)	420 (56.1%)	448 (56.5%)		
Hispanic	306 (19.8%)	150 (20.0%)	156 (19.7%)		
Other	44 (2.85%)	21 (2.80%)	23 (2.90%)		
Multiracial	68 (4.41%)	34 (4.54%)	34 (4.29%)		
Age at DNA methylation measure	12.4 (3.07)	9.30 (0.34)	15.4 (0.49)	-	1542
**Maternal Characteristics at Birth**					
Income-to-needs ratio	2.27 (2.49)	2.29 (2.51)	2.25 (2.48)	0.728	1542
Marital status				0.791	1542
Married	365 (23.7%)	180 (24.0%)	185 (23.3%)		
Not married	1177 (76.3%)	569 (76.0%)	608 (76.7%)		
Race/ethnicity				0.998	1542
Non-Hispanic White	274 (17.8%)	133 (17.8%)	141 (17.8%)		
Non-Hispanic Black	902 (58.5%)	437 (58.3%)	465 (58.6%)		
Hispanic	312 (20.2%)	153 (20.4%)	159 (20.1%)		
Other	54 (3.50%)	26 (3.47%)	28 (3.53%)		
City of residence				>0.999	1542
Oakland	114 (7.39%)	57 (7.61%)	57 (7.19%)		
Baltimore	97 (6.29%)	46 (6.14%)	51 (6.43%)		
Detroit	312 (20.2%)	148 (19.8%)	164 (20.7%)		
Newark	51 (3.31%)	27 (3.60%)	24 (3.03%)		
Philadelphia	120 (7.78%)	59 (7.88%)	61 (7.69%)		
Richmond	143 (9.27%)	73 (9.75%)	70 (8.83%)		
Corpus Christi	93 (6.03%)	44 (5.87%)	49 (6.18%)		
Indianapolis	92 (5.97%)	47 (6.28%)	45 (5.67%)		
Milwaukee	81 (5.25%)	38 (5.07%)	43 (5.42%)		
New York	30 (1.95%)	14 (1.87%)	16 (2.02%)		
San Jose	79 (5.12%)	40 (5.34%)	39 (4.92%)		
Boston	18 (1.17%)	9 (1.20%)	9 (1.13%)		
Nashville	25 (1.62%)	13 (1.74%)	12 (1.51%)		
Chicago	73 (4.73%)	31 (4.14%)	42 (5.30%)		
Jacksonville	20 (1.30%)	10 (1.34%)	10 (1.26%)		
Toledo	87 (5.64%)	39 (5.21%)	48 (6.05%)		
San Antonio	31 (2.01%)	15 (2.00%)	16 (2.02%)		
Pittsburgh	41 (2.66%)	21 (2.80%)	20 (2.52%)		
Norfolk	35 (2.27%)	18 (2.40%)	17 (2.14%)		
**Air Pollution Exposure (μg/m^3^/day)**					
PM2.5 at birth	27.9 (7.04)	27.8 (7.07)	28.0 (7.02)	0.546	1542
Missing					0
PM10 at birth	15.0 (3.06)	15.0 (3.09)	15.0 (3.03)	0.927	1425
Missing	117 (100%)	59 (100%)	58 (100%)		117
PM2.5 at age 1	25.9 (5.29)	25.8 (5.32)	26.0 (5.27)	0.46	1454
Missing	88 (100%)	39 (100%)	49 (100%)		88
PM10 at age 1	14.6 (3.05)	14.6 (3.06)	14.6 (3.04)	0.892	1452
Missing	90 (100%)	41 (100%)	49 (100%)		90
PM2.5 exposure at age 3	26.7 (7.72)	26.6 (7.77)	26.7 (7.67)	0.812	1405
Missing	137 (100%)	65 (100%)	72 (100%)		137
PM10 exposure at age 3	14.2 (3.28)	14.2 (3.29)	14.3 (3.27)	0.664	1414
Missing	128 (100%)	61 (100%)	67 (100%)		128
**DNA Methylation Score**					
PM2.5 methylation score (raw)	−0.05 (0.75)	−0.09 (0.71)	−0.02 (0.77)	0.058	1542
PM2.5 methylation score (centered)	−0.05 (0.75)	−0.09 (0.71)	−0.02 (0.77)	0.058	1542
PM2.5 methylation score (z-score)	−0.07 (0.52)	−0.08 (0.51)	−0.06 (0.54)	0.538	1542
PM10 methylation score (raw)	−0.08 (0.51)	−0.15 (0.47)	−0.02 (0.55)	<0.001	1542
PM10 methylation score (centered)	−0.08 (0.51)	−0.15 (0.47)	−0.02 (0.55)	<0.001	1542
PM10 methylation score (z-score)	−0.09 (0.22)	−0.12 (0.21)	−0.06 (0.22)	<0.001	1542
NO2 methylation score (raw)	−0.02 (0.89)	−0.05 (0.87)	0.01 (0.91)	0.188	1542
NO2 methylation score (centered)	−0.02 (0.89)	−0.05 (0.87)	0.01 (0.91)	0.188	1542
NO2 methylation score (z-score)	−0.05 (0.71)	−0.05 (0.69)	−0.06 (0.73)	0.734	1542
**Saliva Cell Composition**					
Percent immune cells	93.9 (13.6)	95.3 (11.8)	92.5 (14.9)	<0.001	1542
Percent epithelial cells	6.15 (13.6)	4.69 (11.8)	7.52 (14.9)	<0.001	1542
**Site-Specific DNA Methylation**					
cg00905156	2.48 (1.52)	2.30 (1.40)	2.64 (1.60)	<0.001	1542
cg06849931	73.4 (13.5)	74.8 (12.1)	72.0 (14.5)	<0.001	1542
cg15082635	1.91 (0.77)	1.74 (0.60)	2.07 (0.88)	<0.001	1542
cg18640183	4.82 (1.21)	4.79 (1.21)	4.84 (1.20)	0.380	1542
cg20340716	92.8 (1.37)	92.7 (1.46)	92.9 (1.27)	0.002	1542
cg24127244	2.46 (0.65)	2.34 (0.57)	2.57 (0.71)	<0.001	1542

Footer: Bold indicates category of characteristics described.

**Table 2:** Adjusted associations between the cumulative DNA methylation score for prenatal particulate matter (PM) exposure and levels of prenatal particulate matter exposure in the Fragile Families and Child Wellbeing Study. All age models are mixed-effects regression models with random intercepts for participants. Age-stratified models are linear regression models. Models are adjusted for age at DNA measurement, child sex, maternal race, maternal income-to-needs ratio, proportion of epithelial cells, and proportion of immune cells. Effect estimates and confidence intervals are provided for an interquartile increase in exposure (PM2.5: 10.73 μg/m^3^/day; PM10: 3.20 μg/m^3^/day).

				Raw DNA Methylation				Centered DNA Methylation				Centered and Scaled DNA Methylation			
Exposure	Age	*n*_indiv_	*n*_obs_	Effect Estimate	Lower Confidence Interval	Upper Confidence Interval	*p*-Value	Effect Estimate	Lower Confidence Interval	Upper Confidence Interval	*p*-Value	Effect Estimate	Lower Confidence Interval	Upper Confidence Interval	*p*-Value
PM2.5	All	787	1542	−0.029	−0.073	0.016	0.206	−0.029	−0.073	0.016	0.206	−0.017	−0.051	0.018	0.345
PM2.5	9	749	749	−0.021	−0.070	0.028	0.399	−0.021	−0.070	0.028	0.399	−0.014	−0.054	0.026	0.478
PM2.5	15	793	793	−0.017	−0.065	0.030	0.475	−0.017	−0.065	0.030	0.475	−0.008	−0.047	0.031	0.675
PM10	All	728	1425	−0.147	−0.304	0.010	0.066	−0.147	−0.304	0.010	0.066	−0.133	−0.274	0.008	0.065
PM10	9	690	690	−0.004	−0.023	0.015	0.701	−0.004	−0.023	0.015	0.701	−0.005	−0.021	0.012	0.573
PM10	15	735	735	−0.024	−0.043	−0.005	0.012	−0.024	−0.043	−0.005	0.012	−0.023	−0.039	−0.007	0.005

*n*_indiv_ represents the sample size of individual participants. *n*_obs_ represents the number of DNA methylation observations. This number of observations may exceed the sample size of individuals when repeated measures are used.

**Table 3:** Adjusted associations between single DNA methylation sites and levels of prenatal particulate matter (10 µM) exposure in the three months prior to birth in the Fragile Families and Child Wellbeing Study. Multivariable linear regression models have been adjusted for age at DNA measurement, child sex, maternal race, maternal income-to-needs ratio, proportion of epithelial cells, and proportion of immune cells. The sites were measured on the Illumina 450 k array, and the proportions of cells were estimated from the array (*n* = 735). Effect estimates and confidence intervals are for an interquartile range increase in exposure (3.20 µg/m^3^/day). Published cord blood DNA methylation is selected based on prior evidence of association with air pollution [10]. Multivariable linear regression models have been adjusted for age at DNA measurement, child sex, maternal race, maternal income-to-needs ratio, proportion of epithelial cells, and proportion of immune cells (*n* = 735). Effect estimates and confidence intervals are for an interquartile range increase in exposure (3.20 µg/m^3^/day). Published cord blood DNA methylation is from [10].

				Saliva DNA Methylation Age 15 in the Fragile Families and Child Wellbeing Study				Published Cord Blood DNA Methylation	
DNA Methylation Site	Nearest Gene	Chr	Position	Effect Estimate	Lower Confidence Interval	Upper Confidence Interval	*p*-Value	Effect Estimate	*p*-Value
cg00905156	*FAM13A*	4	89744363	−0.048	−0.191	0.094	0.506	0.001	3.55 × 10^−7^
cg06849931	*NOTCH4*	6	32165893	0.160	−0.228	0.547	0.420	−0.001	1.72 × 10^−6^
cg15082635	*GNB2L1*; *SNORD96A*	5	180670110	−0.009	−0.085	0.068	0.821	0.001	8.29 × 10^−8^
cg18640183	*P4HA2*	5	131563610	−0.119	−0.224	−0.014	0.027	0.001	1.80 × 10^−6^
cg20340716	*USP43*	17	9559558	0.135	0.026	0.244	0.015	−0.002	1.50 × 10^−7^
cg24127244	*SRPRB*	3	133524572	−0.015	−0.076	0.046	0.627	0.001	7.33 × 10^−7^

Chr represents the chromosome number.

## Data Availability

Data for the Fragile Families and Child Wellbeing Study are publicly available (https://fragilefamilies.princeton.edu/data-and-documentation/public-data-documentation). Restricted use data, including residential information, are available to researchers through a Contract Data Agreement ensuring Institutional Review Board approval and data protection plans (https://fragilefamilies.princeton.edu/restricted). Code to produce the analyses presented are available (https://github.com/bakulskilab).

## Supplementary Materials

The following are available online at https://www.mdpi.com/article/10.3390/toxics9100262/s1. Table S1: Descriptive statistics of the included and excluded study samples from the Fragile Families and Child Wellbeing Study, Table S2: Adjusted associations between DNA methylation score for prenatal particulate matter exposure and levels of prenatal particulate matter exposure in the Fragile Families and Child Wellbeing Study, Table S3: Adjusted associations between DNA methylation score for prenatal particulate matter exposure and levels of prenatal particulate matter exposure in the Fragile Families and Child Wellbeing Study, Table S4: Adjusted associations between DNA methylation score for prenatal particulate matter exposure and levels of prenatal particulate matter exposure in the Fragile Families and Child Wellbeing Study, Table S5: Adjusted associations between DNA methylation score for prenatal nitrogen dioxide exposure and levels of prenatal particulate matter exposure in the Fragile Families and Child Wellbeing Study, Figure S1: Analytic samples by age and particulate matter type in the Fragile Families and Child Wellbeing Study, Figure S2: Distribution of particulate matter exposure levels during the three months prior to birth in the Fragile Families and Child Wellbeing Study, Figure S3: Pearson correlations among air pollution levels (PM2.5, PM10) measured at birth, age 1, and age 3 in the Fragile Families and Child Wellbeing Study, Figure S4: Distribution of air pollution cumulative DNA methylation scores in the Fragile Families and Child Wellbeing Study, Figure S5: Pearson correlations among air pollution cumulative DNA methylation scores in the Fragile Families and Child Wellbeing Study, Figure S6: Among Fragile Families and Child Wellbeing Study participants with saliva DNA methylation measures at both age 9 and age 15, scatter plots of the observed DNA methylation measures at each time point, Figure S7: Adjusted associations between particulate matter exposure and cumulative DNA methylation score for particulate matter.
