# Supplementary material for: Prenatal Particulate Matter Exposure Is Associated with Saliva DNA Methylation at Age 15: Applying Cumulative DNA Methylation Scores as an Exposure Biomarker
Source: Toxics. 2021 Oct 13;9(10):262. doi: 10.3390/toxics9100262 (PMC8538839; doi:10.3390/toxics9100262)
Supplement: Supplementary file 1 [file toxics-09-00262-s001.zip › toxics-1342332-supplementary.pdf]

toxics MDPI

Supplementary Materials: Prenatal Particulate Matter Exposure Is Associated with Saliva DNA Methylation at Age 15: Applying Cumulative DNA Methylation Scores as an Exposure Biomarker

Kelly M. Bakulski, Jonah D. Fisher, John F. Dou, Arianna Gard, Lisa Schneper, Daniel A. Notterman, Erin B. Ware and Colter Mitchell

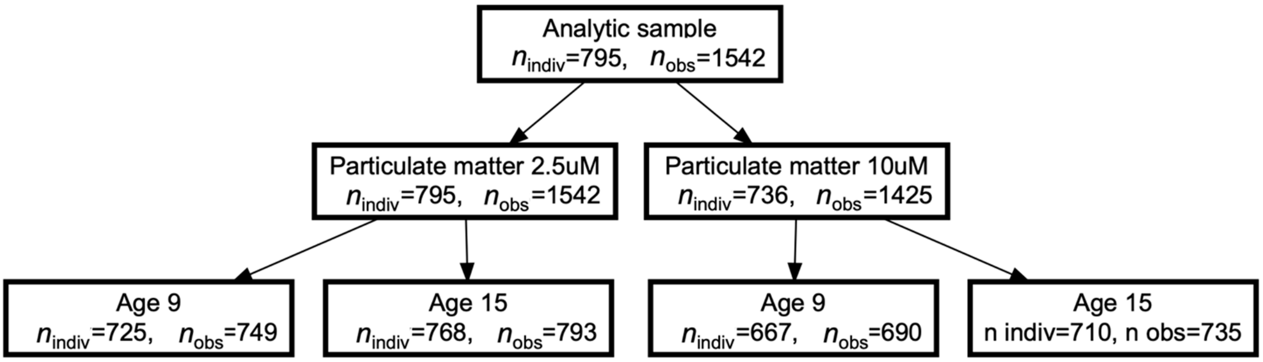

Figure S1. Analytic samples by age and particulate matter type in the Fragile Families and Child Wellbeing Study. n_{indiv} represents the sample size of individual participants. n_{obs} represents the number of DNA methylation observations. The number of observations can exceed the participants when repeated measures are available.

Table S1. Descriptive statistics of the included and excluded study samples from the Fragile Families and Child Wellbeing study.

	**Overall** *n* = 1811	**Excluded sample** *n* = 269	**Included sample** *n* = 1542	***p*-value**	***n***
Age at visit				0.517	1811
Age 9	886 (48.9%)	137 (50.9%)	749 (48.6%)		
Age 15	925 (51.1%)	132 (49.1%)	793 (51.4%)		
Child sex				0.688	1811
Female	899 (49.6%)	130 (48.3%)	769 (49.9%)		
Male	912 (50.4%)	139 (51.7%)	773 (50.1%)		
Child race/ethnicity				<0.001	1811
Non-Hispanic White	314 (17.3%)	58 (21.6%)	256 (16.6%)		
Non-Hispanic Black	965 (53.3%)	97 (36.1%)	868 (56.3%)		
Hispanic	410 (22.6%)	104 (38.7%)	306 (19.8%)		
Other	50 (2.76%)	6 (2.23%)	44 (2.85%)		
Multi-Racial	72 (3.98%)	4 (1.49%)	68 (4.41%)		
Age at DNA methylation measure	12.5 (3.09)	12.8 (3.22)	12.4 (3.07)	0.115	1780
Missing	31 (100%)	31 (100%)	0 (0.00%)	.	31
Maternal income/poverty at birth	2.24 (2.45)	2.07 (2.17)	2.27 (2.49)	0.179	1811

	Overall<br>n = 1811	Excluded sample<br>n = 269	Included sample<br>n = 1542	p-value	n
Maternal race/ethnicity				<0.001	1811
Non-Hispanic White	336 (18.6%)	62 (23.0%)	274 (17.8%)		
Non-Hispanic Black	999 (55.2%)	97 (36.1%)	902 (58.5%)		
Hispanic	416 (23.0%)	104 (38.7%)	312 (20.2%)		
Other	60 (3.31%)	6 (2.23%)	54 (3.50%)		
Maternal marital status				0.579	1807
Married	423 (23.4%)	58 (21.9%)	365 (23.7%)		
Not Married	1384 (76.6%)	207 (78.1%)	1177 (76.3%)		
Missing	4 (100%)	4 (100%)	0 (0.00%)	.	4
Maternal city of residence at birth				.	1811
Oakland	116 (6.41%)	2 (0.74%)	114 (7.39%)		
Austin	127 (7.01%)	127 (47.2%)	0 (0.00%)		
Baltimore	107 (5.91%)	10 (3.72%)	97 (6.29%)		
Detroit	335 (18.5%)	23 (8.55%)	312 (20.2%)		
Newark	56 (3.09%)	5 (1.86%)	51 (3.31%)		
Philadelphia	125 (6.90%)	5 (1.86%)	120 (7.78%)		
Richmond	160 (8.83%)	17 (6.32%)	143 (9.27%)		
Corpus Christi	112 (6.18%)	19 (7.06%)	93 (6.03%)		
Indianapolis	93 (5.14%)	1 (0.37%)	92 (5.97%)		
Milwaukee	87 (4.80%)	6 (2.23%)	81 (5.25%)		
New York	36 (1.99%)	6 (2.23%)	30 (1.95%)		
San Jose	84 (4.64%)	5 (1.86%)	79 (5.12%)		
Boston	19 (1.05%)	1 (0.37%)	18 (1.17%)		
Nashville	27 (1.49%)	2 (0.74%)	25 (1.62%)		
Chicago	82 (4.53%)	9 (3.35%)	73 (4.73%)		
Jacksonville	22 (1.21%)	2 (0.74%)	20 (1.30%)		
Toledo	102 (5.63%)	15 (5.58%)	87 (5.64%)		
San Antonio	38 (2.10%)	7 (2.60%)	31 (2.01%)		
Pittsburgh	48 (2.65%)	7 (2.60%)	41 (2.66%)		
Norfolk	35 (1.93%)	0 (0.00%)	35 (2.27%)		
PM2.5 exposure at birth	28.0 (7.04)	29.7 (6.77)	27.9 (7.04)	0.018	1634
Missing	177 (100%)	177 (100%)	0 (0.00%)	.	177
PM10 exposure at birth	15.0 (3.05)	14.9 (2.89)	15.0 (3.06)	0.808	1515
Missing	296 (100%)	179 (100%)	117 (100%)	.	296
PM2.5 exposure at age 1	25.7 (5.29)	24.0 (4.91)	25.9 (5.29)	<0.001	1677
Missing	134 (100%)	46 (100%)	88 (100%)	.	134
PM10 exposure at age 1	14.3 (3.16)	12.3 (3.10)	14.6 (3.05)	<0.001	1677
Missing	134 (100%)	44 (100%)	90 (100%)	.	134
PM2.5 exposure at age 3	26.4 (7.56)	24.4 (6.12)	26.7 (7.72)	<0.001	1627

	Overall n = 1811	Excluded sample n = 269	Included sample n = 1542	p-value	n
Missing	184 (100%)	47 (100%)	137 (100%)	.	184
PM10 exposure at age 3	14.0 (3.27)	12.4 (2.80)	14.2 (3.28)	<0.001	1636
Missing	175 (100%)	47 (100%)	128 (100%)	.	175
PM2.5 methylation score (raw)	0.00 (1.00)	0.30 (1.86)	-0.05 (0.75)	0.003	1811
PM10 methylation score (raw)	0.00 (1.00)	0.48 (2.23)	-0.08 (0.51)	<0.001	1811
NO2 methylation score (raw)	0.00 (1.00)	0.10 (1.47)	-0.02 (0.89)	0.207	1811
Percent saliva immune cells	93.7 (13.7)	92.6 (14.2)	93.9 (13.6)	0.187	1811
Percent saliva epithelial cells	6.33 (13.7)	7.38 (14.2)	6.15 (13.6)	0.187	1811
Site specific DNA methylation					
cg00905156	2.69 (3.23)	3.89 (7.45)	2.48 (1.52)	0.002	1811
cg06849931	73.1 (13.6)	71.2 (14.1)	73.4 (13.5)	0.019	1811
cg15082635	2.16 (3.59)	3.59 (9.00)	1.91 (0.77)	0.002	1811
cg18640183	5.20 (4.06)	7.37 (9.86)	4.82 (1.21)	<0.001	1811
cg20340716	92.4 (3.95)	90.3 (9.45)	92.8 (1.37)	<0.001	1811
cg24127244	2.68 (3.00)	3.94 (7.52)	2.46 (0.65)	0.001	1811

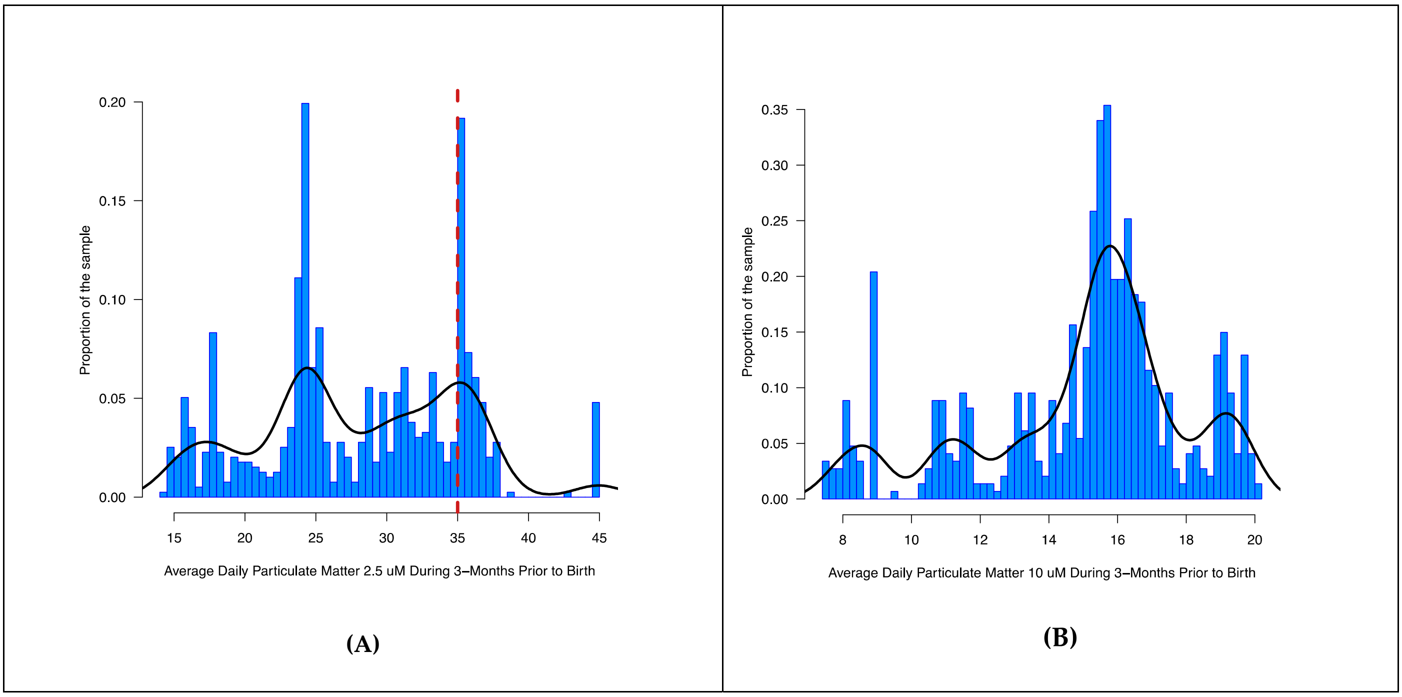

Figure S2. Distribution of particulate matter exposure levels during the 3-months prior to birth in the Fragile Families and Child Wellbeing Study. **A**) Particulate matter < 2.5 µM levels measured in µg/m3/day. The red vertical line corresponds to the United States National Ambient Air Quality Standard (https://www.epa.gov/criteria-air-pollutants/naaqs-table). **B**) Particulate matter < 10 µM levels measured in µg/m^3/day.

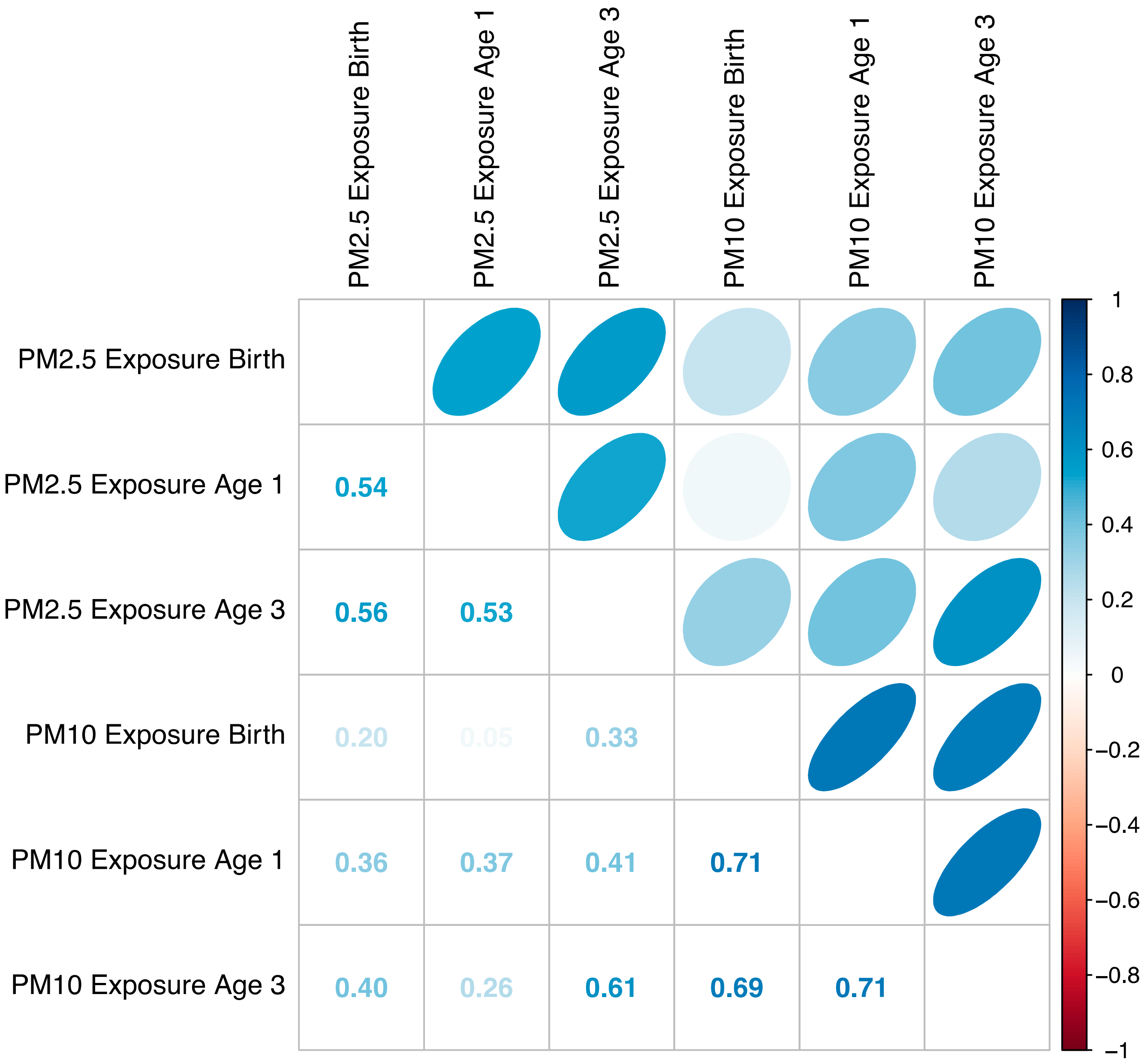

Figure S3. Pearson correlations among air pollution levels (PM2.5, PM10) measured at birth, age 1, and age 3 in the Fragile Families and Child Wellbeing Study.

Pollutant	Raw DNA methylation scores	Probe mean centered DNA methylation scores	Probe z-score standardized DNA methylation scores
**PM2.5**	**A.** 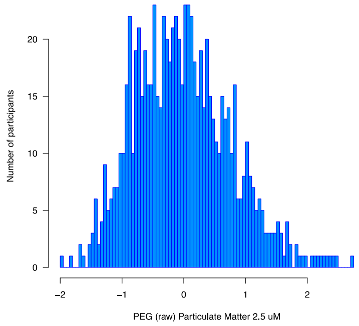	**B.** 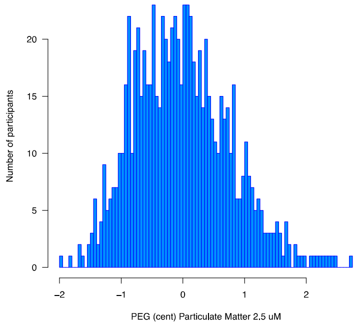	**C.** 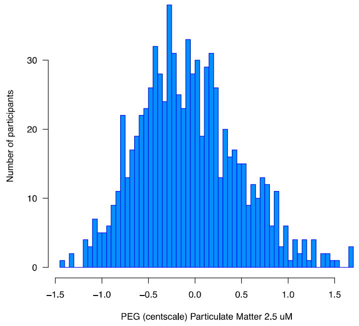
**PM10**	**D.** 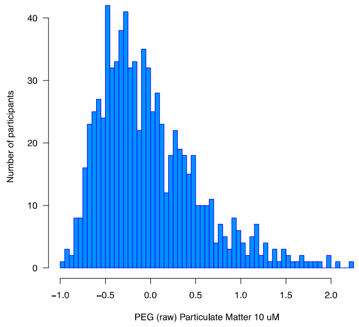	**E.** 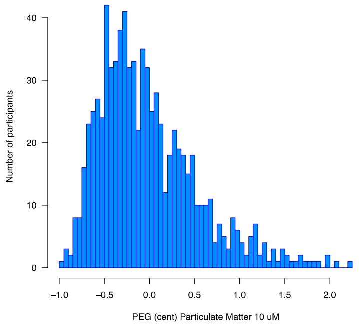	**F.** 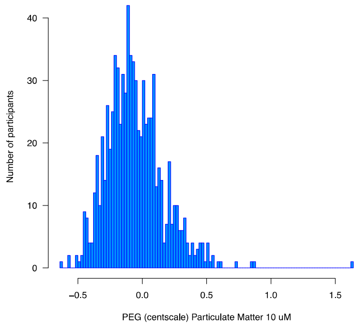
**NO2**	**G.** 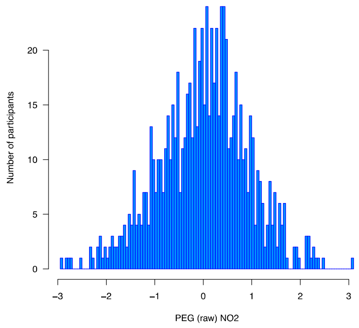	**H.** 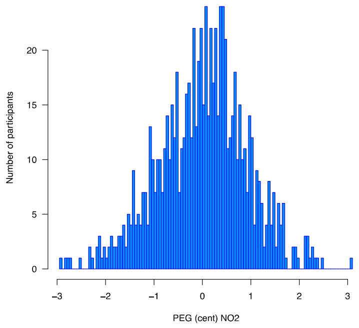	**I.** 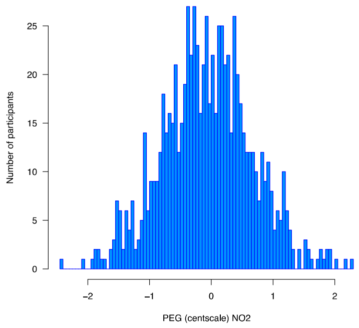

Figure S4. Distribution of air pollution cumulative DNA methylation scores in the Fragile Families and Child Wellbeing Study. The rows are organized by pollutant: **A**, **B**, **C**) Particulate Matter < 2.5 uM; **D**, **E**, **F**) Particulate Matter < 10 uM; **G**, **H**, **I**) Nitrogen dioxide. The columns are organized by method of calculation of cumulative DNA methylation scores. **A**, **D**, **G**) Raw DNA methylation values were used. **B**, **E**, **H**) DNA methylation values were centered within the study sample prior to weighting for the scores. **C**, **F**, **I**) DNA methylation values were centered and scaled within the study sample prior to weighting for the scores.

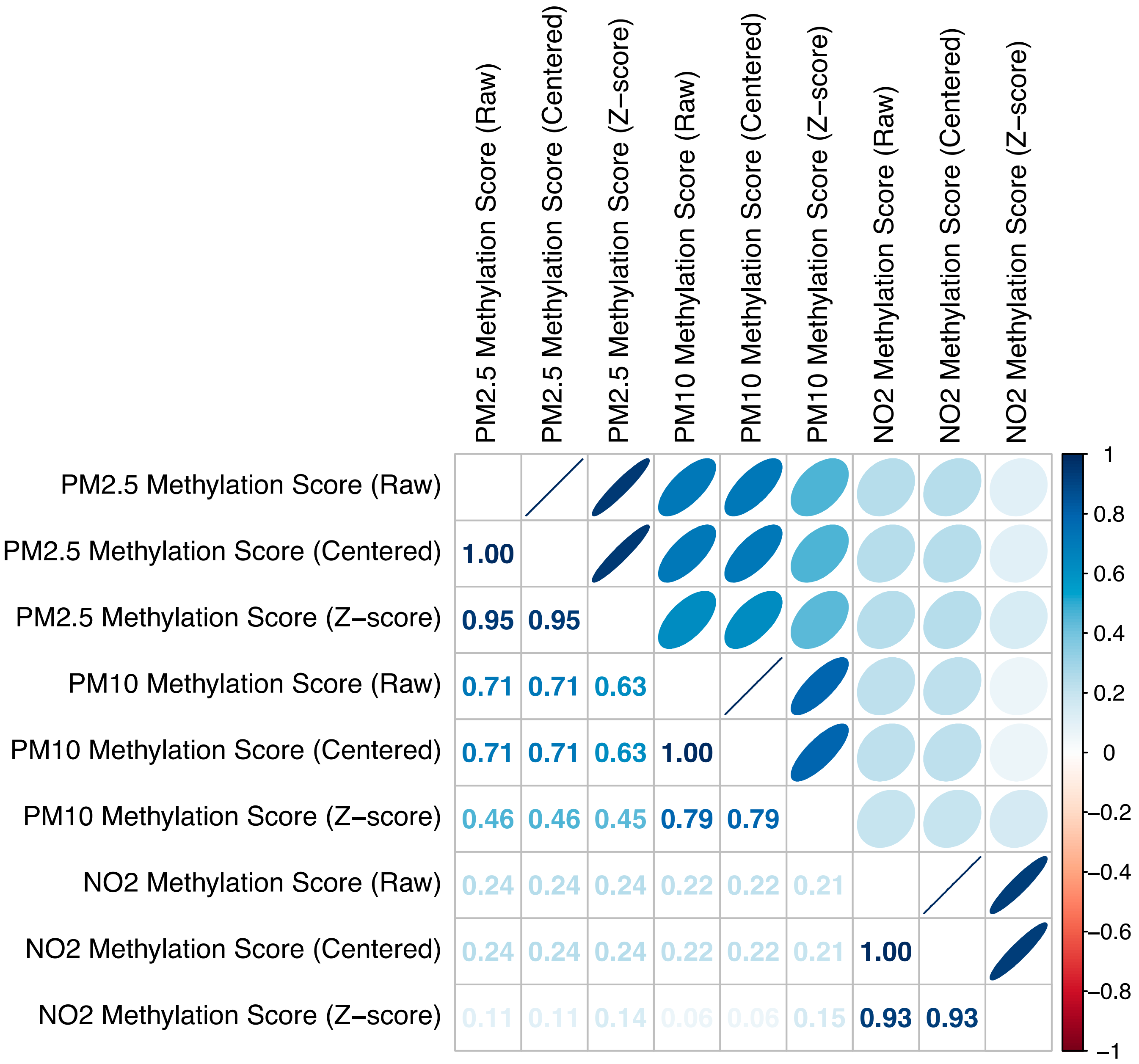

Figure S5. Pearson correlations among air pollution cumulative DNA methylation scores in the Fragile Families and Child Wellbeing Study.

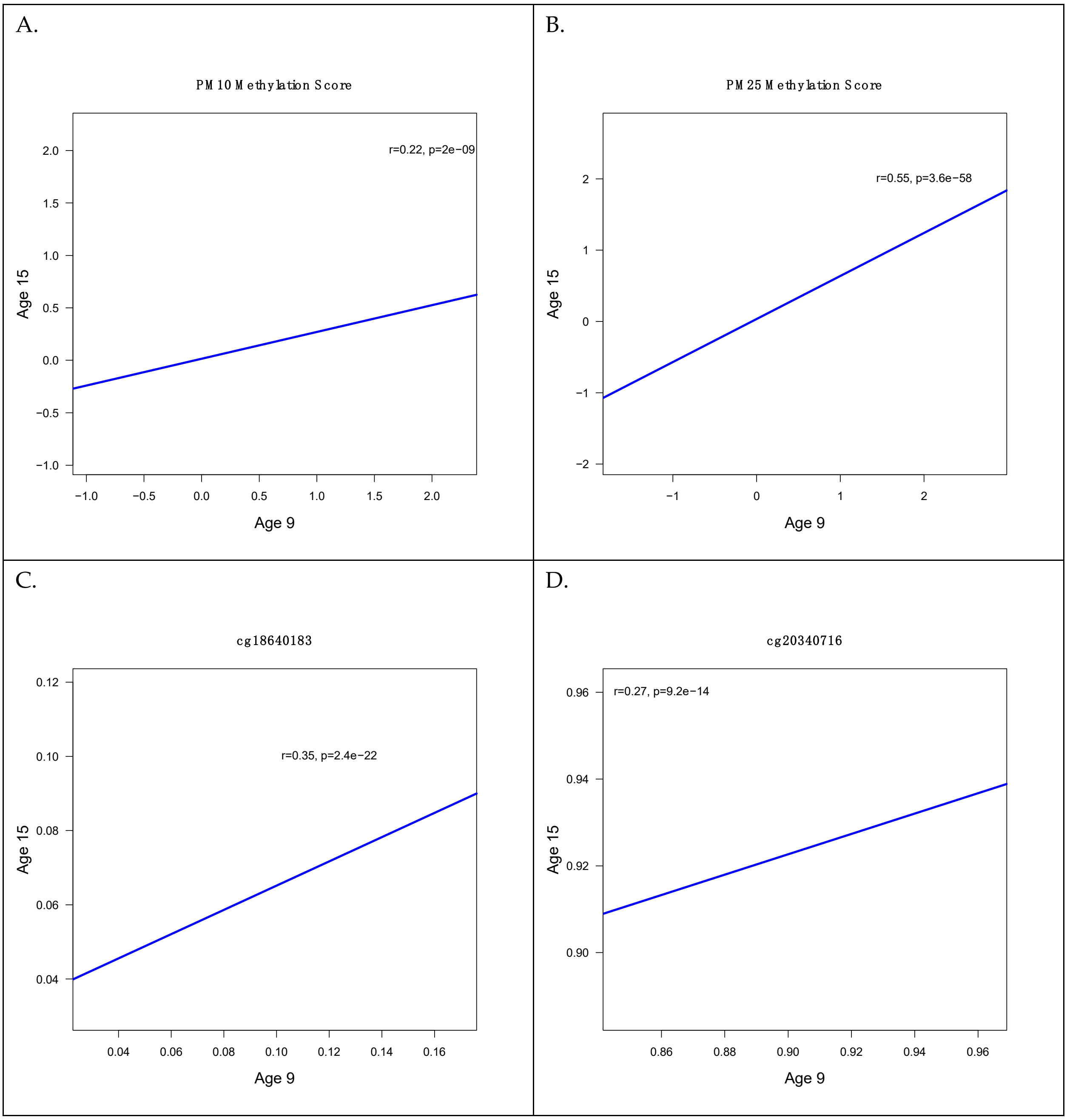

Figure S6. Among Fragile Families and Child Wellbeing participants with saliva DNA methylation measures at both age 9 and age 15, scatter plots of the observed DNA methylation measures at each time point. Blue lines represent the best fit of the data and test statistics reflect the Pearson correlation coefficient and p-value. **A**) Cumulative DNA methylation score (centered and standardized) for PM10 exposure. **B**) Cumulative DNA methylation score (centered and standardized) for PM2.5 exposure. **C**) DNA methylation at the site cg18640183, previously identified (Gruzieva et al. 2019) for association with PM10 exposure at birth. **D**) DNA methylation at the site cg20340716, previously identified (Gruzieva et al. 2019) for association with PM10 exposure at birth.

<table>
<tr><th>Pollutant</th><th>All participants and observations</th><th>Age 9</th><th>Age 15</th></tr>
<tr><td>PM2.5</td><td>A.
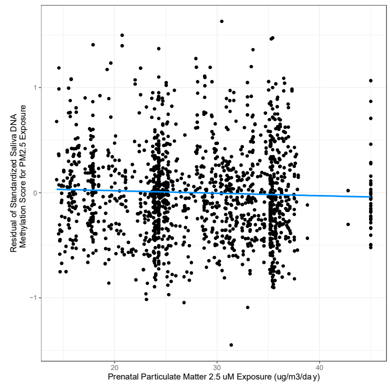
</td><td>B.
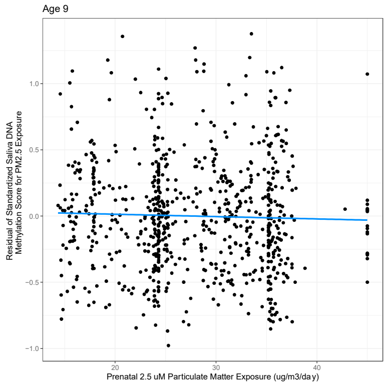
</td><td>C.
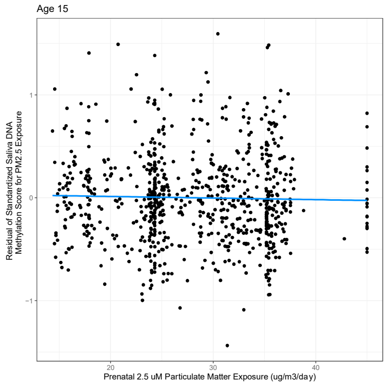
</td></tr>
<tr><td>PM10</td><td>D.
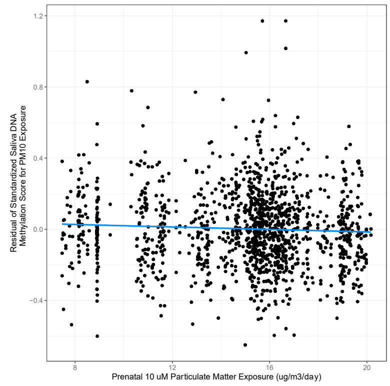
</td><td>E.
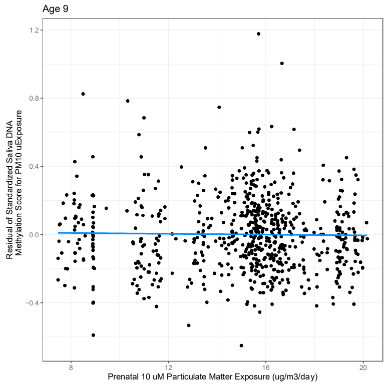
</td><td>F.
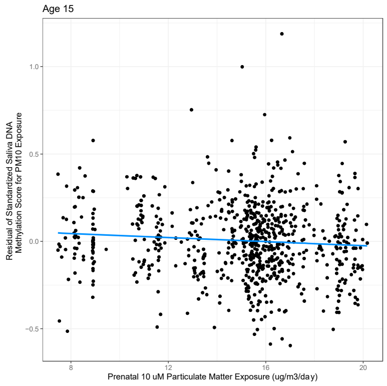
</td></tr>
</table>

Figure S7. Adjusted associations between particulate matter exposure and cumulative DNA methylation score for particulate matter. Models have been adjusted for child sex, child age at DNA methylation measure, maternal income to needs ratio, maternal marital status, maternal race/ethnicity, and cell type proportions.

Table S2. Adjusted associations between DNA methylation score for prenatal particulate matter exposure and levels of prenatal particulate matter exposure in the Fragile Families and Child Wellbeing Study. All ages models are mixed effects regression models with random intercepts for participants. Age stratified models are linear regression models. Models are adjusted for age at DNA measurement, child sex, maternal race, maternal income to poverty ratio, proportion epithelial cells, proportion immune cells, and postnatal age 1 air pollution exposure.

				Raw DNA methylation				Centered DNA methylation				Ce
Exposure	Age	N_{indiv}	N_{obs}	Effect estimate	Lower confidence interval	Upper confidence interval	P-value	Effect estimate	Lower confidence interval	Upper confidence interval	P-value	E est
PM2.5	All	738	1454	-0.0173	-0.0706	0.0360	0.5234	-0.0173	-0.0706	0.0360	0.5234	-0
PM2.5	9	710	710	-0.0101	-0.0682	0.0480	0.7323	-0.0101	-0.0682	0.0480	0.7323	-0
PM2.5	15	744	744	-0.0058	-0.0630	0.0513	0.8411	-0.0058	-0.0630	0.0513	0.8411	0.
PM10	All	682	1342	-0.2422	-0.4546	-0.0298	0.0255	-0.2422	-0.4546	-0.0298	0.0255	-0
PM10	9	653	653	-0.0143	-0.0405	0.0118	0.2828	-0.0143	-0.0405	0.0118	0.2828	-0
PM10	15	689	689	-0.0302	-0.0556	-0.0047	0.0202	-0.0302	-0.0556	-0.0047	0.0202	-0

* N_{indiv} represents the sample size of individual participants

* N_{obs} represents the number of DNA methylation observations. This number of observations may exceed the sample size of individuals when repeated measures are used.

Table S3. Adjusted associations between DNA methylation score for prenatal particulate matter exposure and levels of prenatal particulate matter exposure in the Fragile Families and Child Wellbeing Study. All ages models are mixed effects regression models with random intercepts for participants. Age stratified models are linear regression models. Models are adjusted for age at DNA measurement, child sex, maternal race, maternal income to poverty ratio, proportion epithelial cells, proportion immune cells, and postnatal age 3 air pollution exposure.

				Raw DNA methylation				Centered DNA methylation				C
Exposure	Age	N_{indiv}	N_{obs}	Effect estimate	Lower confidence interval	Upper confidence interval	P-value	Effect estimate	Lower confidence interval	Upper confidence interval	P-value	es
PM2.5	All	714	1405	-0.0316	-0.0893	0.0261	0.2821	-0.0316	-0.0893	0.0261	0.2821	-
PM2.5	9	684	684	-0.0165	-0.0793	0.0463	0.6057	-0.0165	-0.0793	0.0463	0.6057	-
PM2.5	15	721	721	-0.0268	-0.0883	0.0347	0.3929	-0.0268	-0.0883	0.0347	0.3929	-
PM10	All	666	1309	-0.2004	-0.4204	0.0196	0.0742	-0.2004	-0.4204	0.0196	0.0742	-
PM10	9	635	635	-0.0036	-0.0303	0.0231	0.7930	-0.0036	-0.0303	0.0231	0.7930	-
PM10	15	674	674	-0.0343	-0.0604	-0.0082	0.0100	-0.0343	-0.0604	-0.0082	0.0100	-

* N_{indiv} represents the sample size of individual participants

* N_{obs} represents the number of DNA methylation observations. This number of observations may exceed the sample size of individuals when repeated measures are used.

Table S4. Adjusted associations between DNA methylation score for prenatal particulate matter exposure and levels of prenatal particulate matter exposure in the Fragile Families and Child Wellbeing Study. All ages models are mixed effects regression models with random intercepts for participants. Age stratified models are linear regression models. Models are adjusted for age at DNA measurement, child sex, maternal race, maternal income to poverty ratio, proportion epithelial cells, proportion immune cells, and the co-exposure to particulate matter air pollution (either PM2.5 or PM10).

				Raw DNA methylation				Centered DNA methylation				[illegible]
Exposure	Age	N_{indiv}	N_{obs}	Effect estimate	Lower confidence interval	Upper confidence interval	P-value	Effect estimate	Lower confidence interval	Upper confidence interval	P-value	[illegible]
PM2.5	All	727	1425	-0.0278	-0.0806	0.0249	0.3007	-0.0278	-0.0806	0.0249	0.3007	[illegible]
PM2.5	9	690	690	-0.0225	-0.0781	0.0332	0.4280	-0.0225	-0.0781	0.0332	0.4280	[illegible]
PM2.5	15	735	735	-0.0225	-0.0796	0.0346	0.4387	-0.0225	-0.0796	0.0346	0.4387	[illegible]
PM10	All	727	1425	-0.1474	-0.3079	0.0131	0.0718	-0.1474	-0.3079	0.0131	0.0718	[illegible]
PM10	9	690	690	-0.0043	-0.0239	0.0153	0.6671	-0.0043	-0.0239	0.0153	0.6671	[illegible]
PM10	15	735	735	-0.0231	-0.0424	-0.0038	0.0191	-0.0231	-0.0424	-0.0038	0.0191	[illegible]

* N_{indiv} represents the sample size of individual participants

* N_{obs} represents the number of DNA methylation observations. This number of observations may exceed the sample size of individuals when repeated measures are used.

Table S5. Adjusted associations between DNA methylation score for prenatal nitrogen dioxide exposure and levels of prenatal particulate matter exposure in the Fragile Families and Child Wellbeing Study. All ages models are mixed effects regression models with random intercepts for participants. Age stratified models are linear regression models. Models are adjusted for age at DNA measurement, child sex, maternal race, maternal income to poverty ratio, proportion epithelial cells, and proportion immune cells.

				Raw DNA methylation				Centered DNA methylation				[illegible]
Exposure	Age	N_{indiv}	N_{obs}	Effect estimate	Lower confidence interval	Upper confidence interval	*P*-value	Effect estimate	Lower confidence interval	Upper confidence interval	*P*-value	[illegible]
PM2.5	All	787	1542	0.0292	-0.0551	0.1135	0.4969	0.0292	-0.0551	0.1135	0.4969	[illegible]
PM2.5	9	749	749	0.0518	-0.0433	0.1468	0.2853	0.0518	-0.0433	0.1468	0.2853	[illegible]
PM2.5	15	793	793	0.0306	-0.0642	0.1254	0.5265	0.0306	-0.0642	0.1254	0.5265	[illegible]
PM10	All	728	1425	1.4166	0.7019	2.1314	0.0001	1.4166	0.7019	2.1314	0.0001	[illegible]
PM10	9	690	690	0.1386	0.0678	0.2095	0.0001	0.1386	0.0678	0.2095	0.0001	[illegible]
PM10	15	735	735	0.1271	0.0520	0.2022	0.0009	0.1271	0.0520	0.2022	0.0009	[illegible]

* N_{indiv} represents the sample size of individual participants

* N_{obs} represents the number of DNA methylation observations. This number of observations may exceed the sample size of individuals when repeated measures are use
